# Integrated Mechano-Electrochemical Harvesting Fiber and Thermally Responsive Artificial Muscle for Self-Powered Temperature–Strain Dual-Parameter Sensor

**DOI:** 10.3390/s23010269

**Published:** 2022-12-27

**Authors:** Hyeon Jun Sim, Jun Ho Noh, Jin Hyeong Choi, Changsoon Choi

**Affiliations:** 1 Department of Energy and Materials Engineering, Dongguk University, Seoul 04620, Republic of Korea; 2 Department of Advanced Battery Convergence Engineering, Dongguk University, Seoul 04620, Republic of Korea; 3 Research Center for Photoenergy Harvesting & Conversion Technology (PHCT), Dongguk University, Seoul 04620, Republic of Korea

**Keywords:** mechano-electrochemical energy harvester, artificial muscle, self-powered, strain–temperature sensor, soft, fiber, stretchable

## Abstract

Significant progress in healthcare fields around the world has inspired us to develop a wearable strain–temperature sensor that can monitor biomedical signals in daily life. This novel self-powered temperature–strain dual-parameter sensor comprises a mechano-electrochemical harvester (MEH) and a thermally responsive artificial muscle (TAM). The MEHTAM system generates electricity from strain and thermal fluctuations. In addition, the sensor is comfortable to wear, owing to its stretchability (>100%), softness (<3 MPa), and one-dimensional fibers (diameter 230 μm). The MEH induces a change in the electrochemical capacitance, resulting in an electrical signal under applied strain (34 μA/m) and stress (20 μA/(m·MPa)). The TAM can be used as a mechanical temperature sensor, because the tensile stroke responds linearly to changes in temperature. As the harvester and artificial muscle are combined, the MEHTAM system generates electricity, owing to external and internal mechanical stimuli caused by muscle contractions as a response to temperature changes. The MEHTAM system that we have developed—a self-powered, strain–temperature dual-parameter sensor that is soft, stretchable, and fiber-shaped—is an interesting candidate for the production of comfortable, wearable, dual-parameter sensors.

## 1. Introduction

There has been great progress in healthcare fields worldwide during the last decade, which has inspired us to develop a wearable sensor that can monitor environmental, fitness, and medical data [1,2,3]. Researchers have devoted extensive efforts toward developing devices that can detect biomedical signals, including pressure/strain, temperature, and biochemical [4,5,6,7,8]. Multifunctional sensors have simpler structures, and cost less when forming multi-parametric sensing systems: in particular, self-powered sensors that do not require an external power source have been extensively researched in academic and industrial fields [4,5,6].

Generally, sensors have only detected single signals. Several recent approaches to multifunctional sensors, with the ability to detect two pieces of information by measuring two signals, have been reported [4,5]: however, they were relatively unstable, compared to measuring one signal. Although multifunctional sensors experience difficulties in distinguishing different signals simultaneously, their purpose is to expand sensing capabilities [8]. With advances in wearable human monitoring systems based on the Internet of Things, numerous sensors are required for various measurements, perceptions, controls, and data transmission: however, these randomly and massively distributed sensing networks require energy sources to drive the sensors, which are bulky, rigid, brittle, and complex, limiting wearability. To overcome this limitation, self-powered sensors have become a focus in academic and industrial fields [4,5,6]. Self-powered sensors convert environmental energy directly into electric energy, to drive themselves without any external power source, leading to miniaturization and the simplification of complex systems. 

Simultaneously detecting strain and temperature is a crucial aspect of monitoring human activities in daily life [9,10,11,12,13]; therefore, researchers have developed self-powered multifunctional sensors, by combining thermal energy harvesters with mechanical strain sensors or mechanical energy harvesters. Wen et al. fabricated a self-powered strain–temperature dual-functional sensor that responded to resistance changes caused by strain and thermoelectric effects [9]; however, the disadvantage of their device was that an energy source was still required when measuring strain through resistance changes. Wu et al. fabricated a triboelectric–thermoelectric hybrid nanogenerator [12]; however, brittle, substrate-based two-dimensional (2D) disk structures are difficult to apply in wearable systems; furthermore, sensors made of nonporous film can irritate the skin, making it red and itchy [9]: hence, a new approach was needed, to eliminate these deficiencies.

In this study, we designed a novel self-powered temperature–strain dual-parameter sensor that combines two devices: a mechano-electrochemical harvester (MEH), which converts mechanical energy into electrical energy through an electrochemical principle; and a thermally responsive artificial muscle (TAM), which converts thermal energy into mechanical energy. This MEHTAM system generates electricity from strain and thermal fluctuations due to contractions of the artificial muscle. The stretchability (>100%), softness (<3 MPa), and 1D fibers (diameter of 230 μm) render the system comfortable to wear; moreover, the fiber is flexible, and can be woven into a 2D textile, solving the permeability problem of nonporous film-type sensors.

The MEH is composed of a carbon nanotube (CNT) sheet with microscale buckles, which is wrapped around an elastomeric fiber: this induces a change in the electrochemical capacitance, which results in an electrical signal under applied strain (34 μA/m) and stress (20 μA/(m·MPa)). The TAM is a strongly coiled nylon thread, which can be used as a mechanical temperature sensor, because the tensile stroke is linearly dependent on temperature. When the harvester and artificial muscle are combined, the MEHTAM system generates electricity, owing to external and internal mechanical stimuli caused by muscle contractions according to the temperature. The soft and stretchable self-powered strain–temperature dual-parameter sensor with 1D fibers is comfortable to wear. Furthermore, the novel design can promote the development of self-powered dual-parameter sensors, such as strain–humidity, strain–photo, strain–chemistry, and strain–biochemistry.

## 2. Materials and Methods

The buckled CNT sheet/elastomer fiber was fabricated using highly aligned CNTs, which were grown on silicon wafers via chemical vapor deposition. Two-end-tethered spandex fibers (average diameter of approximately 220 μm, and length of 4 cm) were prepared for the pre-strain process, and were then stretched by 400% in the tensile direction. Subsequently, a pre-strained fiber was wrapped in a CNT sheet, which was aligned along the length of the elastomeric fiber. In the next step, the fiber was compressed with ethanol, and dried. Finally, the CNT-wrapped elastomeric fiber was released, and it contracted by itself. The nylon fiber was coiled by twisting it many times. More specifically, one end of a 102-μm-diameter nylon 66 fiber (D67, Coats & Clark) was loaded with 100 g, and the other end was attached to a motor. The hanging weight was tethered, to prevent rotation: thus, only rotation of the motor twisted the fiber. An appropriate applied weight was selected, because an insufficient weight would have allowed the formation of snarling (entanglement), and excessive weight would have broken the fiber. The MEHTAM system was fabricated by attaching one end of the MEH to one end of the TAM. For maximum tensile actuation of the TAM, a paddle was attached to one of its ends, to prevent rotation. To compress the TAM length, a 120 g load was applied. By using an electrochemical measurement system (working electrode: MEH; counter electrode: Pt mesh; reference electrode: Ag/AgCl), the MEH part was immersed into an electrolyte (saline, 0.6 M NaCl), whereas the TAM part was left outside. In the next step, the TAM part was heated, using a heat gun. To confirm the amount of electricity produced by mechanical stimulation, the electrical output of the MEH was measured while deforming the entire length of the MEHTAM system at a contact temperature. In contrast, when analyzing the electricity produced by thermal stimulation, the electrical output was measured while the entire length of the MEHTAM system was fixed, to restrict any effects of external mechanical stimulation. 

The wrapping process was conducted using a stepper motor (A16K-M569, Autonics, Corp., Busan, Korea). The performance of the MEH was measured with an electrochemical measurement device (model G750, Gamry, Warminster, PA, USA) in a three-electrode arrangement with a working electrode (the MEH), counter electrode (Pt mesh), and a reference electrode (Ag/AgCl). To calculate the electrical power and energy, the electrical output was measured with an external resistance. To ensure reliable results, the voltage was measured with an oscilloscope (DPO4014B, Tektronix, Beaverton, OR, USA), by changing the external resistance. The electrical power (P = V × V/R, where P = power, V = voltage, and R = resistance) was also calculated. Moreover, field-emission SEM (FESEM, Hitachi S4700, Tokyo, Japan) was performed at 15 kV, and a thermal imaging camera (XI 400 macro, Optris, Berline, Deutschland), and an optical camera (SMZ1270, Nikon, Tokyo, Japan) were used to study morphology. The electrical measurements were conducted with a digital multimeter (Model 187, Fluke Corporation, Everett, WA, USA), and the mechanical testing was performed using a universal testing machine (UTM, INSTRON 5966, INSTRON, Norfolk County, MA, USA).

## 3. Results and Discussion

### 3.1. Concept of Combined Mechano-Electrochemical Harvesting Fiber and Thermally Responsive Artificial Muscle

**Figure 1:**
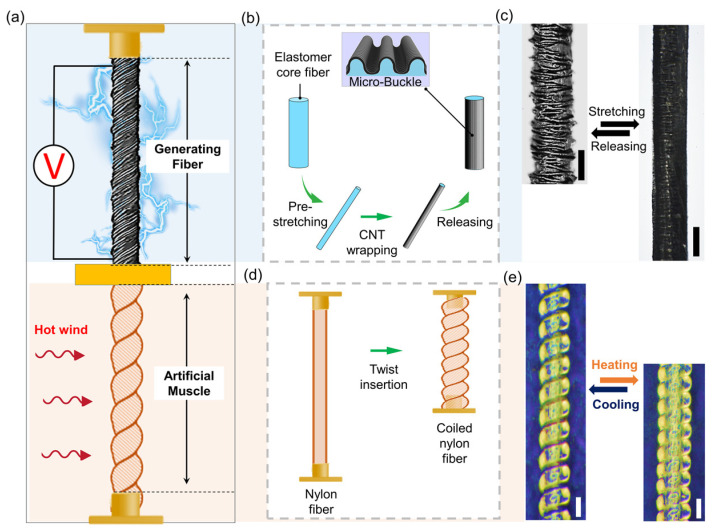
Concept of combined mechano-electrochemical harvesting fiber and thermally responsive artificial muscle: (**a**) schematic diagram of integrated buckled mechano-electrochemical harvesting fiber and coiled artificial muscle structure; (**b**) schematic of fabrication process of mechano-electrochemical harvesting fiber; (**c**) photograph showing elasticity of mechano-electrochemical harvesting fiber (scale bar: 100 µm); (**d**) schematic of fabrication process of coiled artificial muscle (nylon); (**e**) photograph showing thermally responsive actuation of coiled nylon (scale bar: 100 µm).

To design a self-powered temperature–strain dual-parameter sensor, the MEH and TAM devices needed to be developed in advance (Figure 1a). First, the MEH, which converts mechanical into electrical energy, was fabricated (Figure 1b). The elastomeric fiber was stretched to approximately 400% in the tensile direction. Subsequently, the pre-strained elastomeric fiber was wrapped into a CNT sheet. The CNT sheet, in which the CNTs are aligned in the axial direction, has excellent electrical and mechanical conductivity, which makes it a reliable wearable conductor [14]. In the next step, the pre-strained and wrapped fiber contracted to its initial length, which resulted in the spontaneous formation of microscale buckles in the CNT sheet (Supplementary Figure S1). The average diameter of the fibers and the width of the buckles were 230 and 18 μm, respectively. Because the added length of the CNT sheet along the longitudinal direction of the elastomeric fiber was stored in the form of buckles, the fiber became a stretchable conductor. When the fiber was stretched, the number of contact points between the buckles decreased, slightly increasing the resistance. Nevertheless, the electrical pathway in the CNT sheet was maintained while the buckles unfolded (Figure 1c). The electrical resistance ratio, which is the resistance change divided by the initial resistance, remained fairly constant (0.05) during the repeated stretching/releasing cycles (Supplementary Figure S2).

The TAM was then fabricated, to convert thermal into mechanical energy, (Figure 1d). When the nylon fiber was strongly twisted with an adjustable load, a coil formed spontaneously, to minimize the strain energy [15,16]. More specifically, a coiled nylon thread was formed, by twisting 102 µm diameter nylon fiber with a 7.8 MPa load. Subsequently, adjacent coil loops were separated with a load of 9.4 MPa. When the temperature increased, the loops became closer, thereby contracting the TAM (Figure 1e and Supplementary Movie S1).

### 3.2. Generation of Electricity by Mechano-Electrochemical Harvesting Fiber for Self-Powered Strain Sensor 

**Figure 2:**
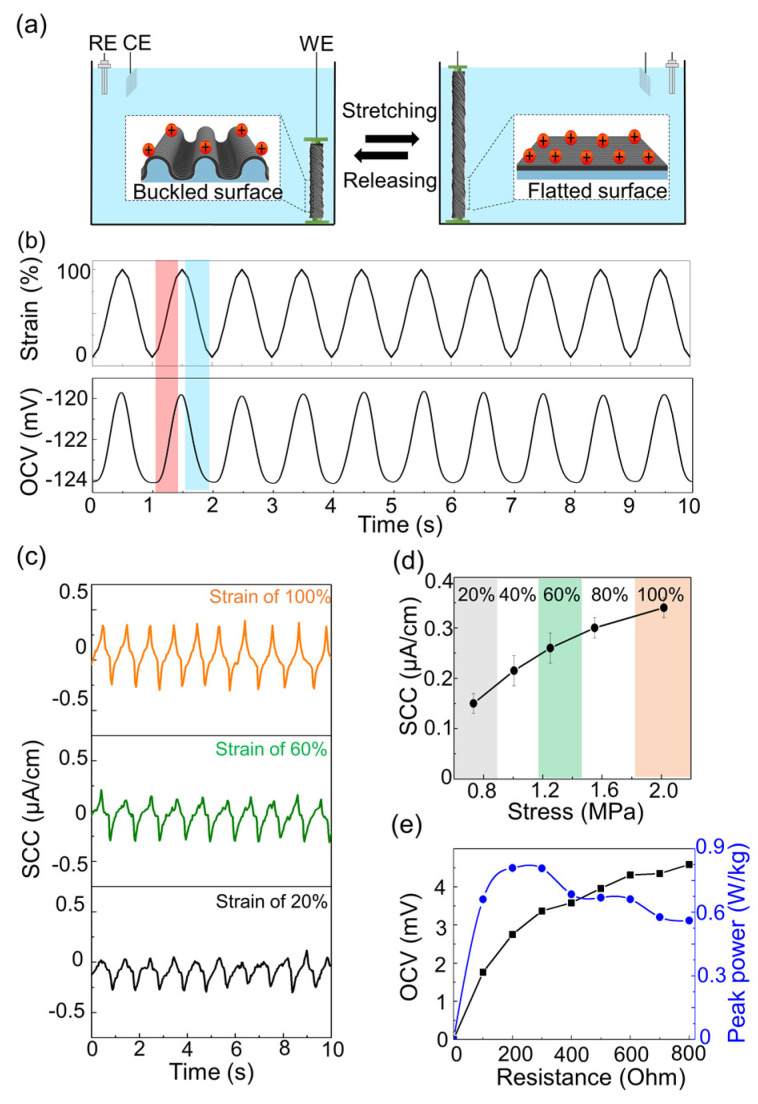
Generation of electricity by mechano-electrochemical harvesting fiber for self-powered strain sensor: (**a**) schematic of electricity generation by buckled fiber with mechanical stretching in electrolyte; (**b**) sinusoidally applied tensile strain results in an open-circuit voltage; red and blue areas denote stretched and released states, respectively; (**c**) short-circuit current at different applied strains, from 20% to 100% during 1 Hz sinusoidal stretch in saline; (**d**) SCC output with applied stress; (**e**) peak-to-peak voltage and peak power of MEH with external load resistance.

The MEH generates electricity based on tensile strain (Figure 2a). After the CNT sheet was immersed in the electrolyte, a potential difference occurred between the surface of the CNT sheet and the surrounding electrolyte (Supplementary Figure S3) [17,18,19]. The potential-induced ion absorption on the electrode surface was in accordance with Q = CV = C(OCV-PZC), where Q represents the charge on the electrode, C is the capacitance of the fiber, V is the intrinsic voltage, OCV is the open-circuit voltage, and PZC is the potential of zero charge.

When the fiber was stretched, the unfolding buckle increased the electrochemical capacitance by increasing the active surface area, which increased the voltage. Consequently, the OCV increased from −124 to −120 mV when the fiber was stretched to a strain of 100% (Figure 2b); furthermore, the voltage returned to its initial level when the initial length was restored, because the active surface area decreased. Like the trend of the output voltage, a short-circuit current (SCC) was continuously induced, owing to strain (Supplementary Figure S4).

The electrical output of the MEH depended on the applied strain, providing a potential in the self-powered strain sensor. To enhance data reliability, the mean value and standard deviation of five repeated results were plotted (Figure 2c). The SCC increased from 0.16 to 0.34 μA/cm when the applied strain increased from 20% to 100%. Similarly, the peak-to-peak voltage increased with the strain (Supplementary Figure S5). The increasing strain induced a larger change in the capacitance, which increased the electrical output. The device was very sensitive to stress, because the MEH was soft. According to the strain–stress curve of the MEH, it could be repeatedly stretched with a low stress of 2.2 MPa at 100% strain, resulting in a low Young’s modulus of 2.2 MPa (Supplementary Figure S6); therefore, the device could measure small tensile stresses (0.7 MPa). This system can be used to build microfiber-type self-powered stress sensors with high sensitivity (0.2 µA/(cm·MPa; Figure 2d and Supplementary Figure S7). In addition, the soft sensor can detect small stress signals, and is a good candidate for wearable and implantable sensors. Young’s modulus of human organs and tissue is between 0.45 and 60 MPa [20]. Young’s modulus of the MEH was similar to (or lower) than these values, allowing the detection of organ motion strain, while minimizing any damage to the organs and tissue. When the generated electricity of the MEH with an external load of the self-powered system was measured, the peak-to-peak voltage increased with external load resistance. Maximum power (1.6 W/kg) was at a load resistance of 300 ohms (Figure 2e). In addition, the electrical output was constant after 1000 cycles of 0.5 Hz sinusoidal stretching to 100% strain. The voltage remained fairly constant, and the voltage retention decreased to values below 7% (Supplementary Figure S8). In addition, the MEH output remained fairly constant, even under temperature changes from 10 to 40 °C (Supplementary Figure S9).

### 3.3. Thermomechanical Actuation of Coiled Artificial Muscle

**Figure 3:**
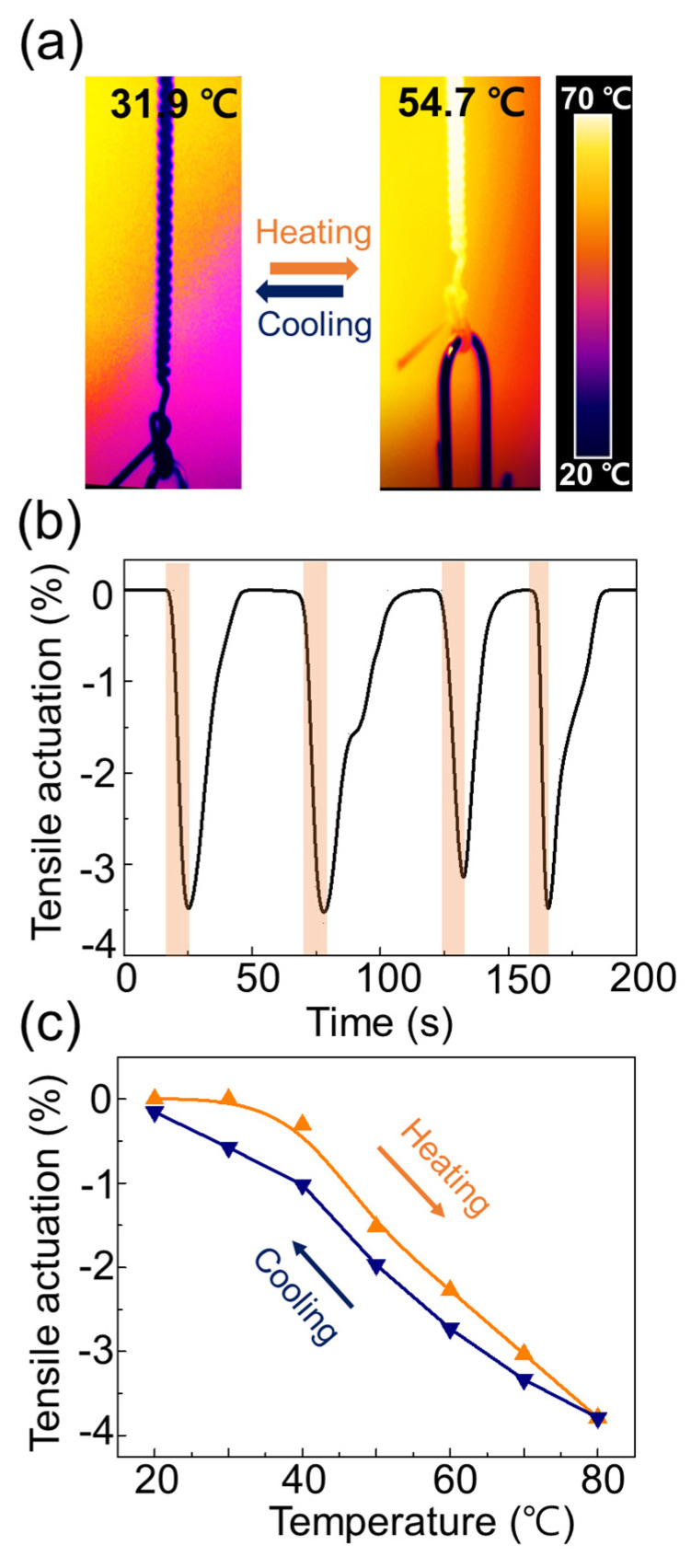
Thermomechanical actuation of coiled artificial muscle: (**a**) infrared thermal images of coiled artificial muscle; (**b**) tensile stroke of coiled nylon when heated by hot air; (**c**) tensile stroke versus temperature of coiled 102 μm diameter nylon monofilament muscle, under 9.4 MPa static load.

The TAM contracted quickly, and provided a large stroke owing to thermal stimulation (Figure 3a). The reversible thermal contraction of the nylon fiber between 20 and 80 °C represented a tensile stroke of −3.5%. The strongly twisted fibers spontaneously formed a coiled structure, to minimize the strain energy: when this two-end-tethered coil was heated, the untwisting motion in the fiber pushed the adjacent coil loops closer together, shortening the TAM. This actuation was caused by thermal expansion of the polymer and force balance of the twisted structure, providing fast and reversible actuation. When a heat flow of 90 °C was applied to the TAM at 26 °C, contractions were repeatedly observed for 8.5 s (Figure 3b). The TAM rapidly returned to its initial length within 25 s, while cooling to room temperature because of the microscale diameter. Moreover, the TAM exhibited linear actuation with respect to temperature (Figure 3c). When the temperature increased from 20 to 80 °C, the coefficient of the tensile stroke was measured, using a linear fitting analysis, and the value decreased from −0.012 to −0.103 %/K^2^ (Supplementary Figure S10). Shape-memory alloys (such as nitinol) have large hysteresis, rendering such devices difficult to control and use as mechanical temperature sensors [15]. Accordingly, linear actuation with low hysteresis allows the TAM to be used as a mechanical thermal sensor.

### 3.4. MEH Fiber with TAM for Self-Powered Strain–Temperature Dual-Parameter Sensor 

**Figure 4:**
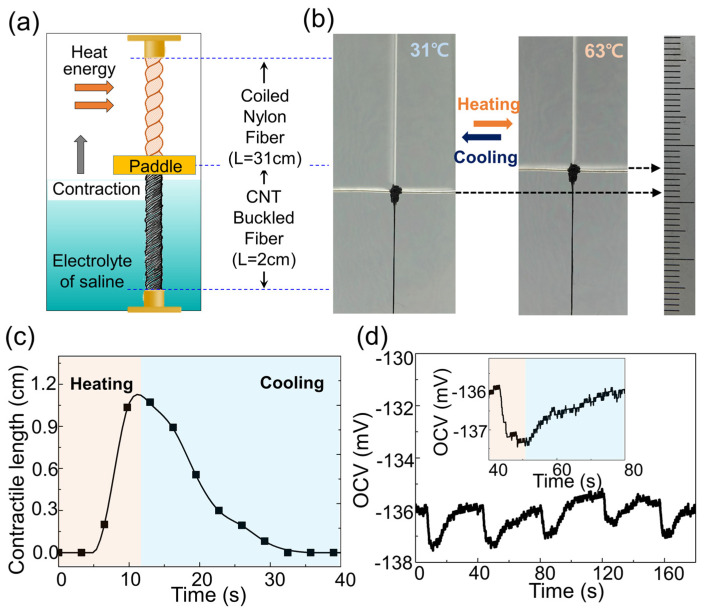
MEH fiber with TAM for self-powered strain–temperature dual-parameter sensor: (**a**) schematic of integrated MEHTAM fiber; (**b**) optical image of MEHTAM system; (**c**) the contraction length and tensile stroke of the TAM in the MEHTAM system were time-dependent when the fiber was being heated or cooled; (**d**) OCV response of MEHTAM system, due to temperature change. Inset: magnified OCV output from 40 to 80 s at extremely low frequency. The red and blue areas correspond to the heating and cooling states of the coiled nylon, respectively.

Owing to its softness, the MEH could be sensitively measured, even under the weak stress exerted by the TAM. Each end of the 2-cm-long MEH and 31-cm-long TAM was fixed with epoxy resin (Figure 4a,b); then, the TAM was subjected to a hot airflow, causing it to contract to approximately 1.1 cm (Figure 4c): thus, the MEH fiber was stretched to 60% strain, which decreased the voltage response (Figure 4d). During the cooling period, the MEH and TAM fibers recovered their initial lengths, which increased the voltage. Therefore, the MEHTAM system is different from existing coupled sensors, because it can detect strain and thermal fluctuations (Supplementary Figure S11). Accordingly, the combination of a harvester and an artificial muscle is suitable for a wide range of devices, including strain–humidity, strain–photonic, strain–chemical, and strain–biochemical sensors. 

## 4. Conclusions

In this study, we built a sensor composed of a mechanical harvester and an artificial muscle. The MEH comprises a buckled CNT sheet on elastomeric fibers. The active surface area, which provides electrochemical capacitance in an electrolyte, changed according to the strain of the fiber, providing electrical signals. The harvester is suitable for wearable sensors, because of its good wearability, due to its softness, high stretchability, and 1D fibers. High stretchability and low Young’s modulus were achieved by transitioning from a coiled to a buckled structure. Fibers with low Young’s modulus can prevent damage to soft organs and tissue, because of their similar softness levels. Moreover, because the microdiameter fibers minimize the contact area at the device–skin interface, the skin does not become irritated (such as when nonporous film-type sensors are used). In addition, no external electrical sources (such as batteries) are required to detect the strain. With regard to the previously presented wearable sensing systems, the energy source eventually had to be recharged or replaced, which is inconvenient in daily life. The self-powered system presented in this paper directly generates electricity from mechanical energy, making recharging unnecessary. The TAM can be constructed with coiled nylon fibers, and this coiled structure stores strain energy via structural deformation. When the fiber untwists due to thermal expansion of the nylon, the adjacent coil loops are pushed closer together, shortening the TAM. The actuation caused by the force balance between the thermal expansion and the twisted structure was fast and reversible. In particular, linear actuation with temperature based on the low hysteresis of the thermally responsive actuation is an advantage of mechanical temperature sensors compared to shape-memory alloys. By combining these two sensor types, a self-powered strain–temperature dual-parameter sensor was developed. The MEHTAM system generates electricity from mechanical and thermal stimulation caused by contraction of the TAM. The combination of a harvester and an artificial muscle is a good candidate for self-powered dual-parameter sensors, such as strain–humidity, strain–photonic, strain–chemical, and strain–biochemical sensors. The performances of the MEH and TAM devices were stable, even under repeated strain. The main challenge facing the proposed sensor is the distinction between mechanical energy and thermal energy through electrical output. We plan to conduct a study to distinguish between thermal and mechanical energy, by adding a resistance change factor.

## Supplementary Materials

The following supporting information can be downloaded at: https://www.mdpi.com/article/10.3390/s23010269/s1, Figure S1: SEM image of MEH fiber; Figure S2: electrical resistance of MEH fiber with strain; Figure S3: OCV value of MEH fiber when the fiber was immersed in the electrolyte of 0.6 M NaCl; Figure S4: sinusoidally applied tensile strain, resulting in short-circuit current value of MEH fiber, when the fiber stretched to 100% strain in an electrolyte of 0.6 M NaCl; Figure S5: open-circuit voltage with various applied strains, from 20% to 100% during 1 Hz sinusoidal stretch in saline; Figure S6: strain–stress curve of MEH fiber; Figure S7: output response of peak-to-peak voltage with applied stress; Figure S8: open-circuit voltage output of fiber after 1000 cycles in saline; Figure S9: The open-circuit peak-to-peak voltage with various environmental temperature from 10 to 40 °C during 1-Hz sinusoidal 100% stretch; Figure S10: the coefficient of tensile stroke with temperature during heating; Figure S11: the SCC of MEH-TAM with temperature; Video S1 of coiled nylon contraction with temperature.
