# Supplementary material for: Integrated Mechano-Electrochemical Harvesting Fiber and Thermally Responsive Artificial Muscle for Self-Powered Temperature–Strain Dual-Parameter Sensor
Source: Sensors (Basel). 2022 Dec 27;23(1):269. doi: 10.3390/s23010269 (PMC9824410; doi:10.3390/s23010269)
Supplement: Supplementary file 1 [file sensors-23-00269-s001.zip › supplementary Figures.pdf]

Supplementary Information

Integrated Mechano-Electrochemical Harvesting Fiber and Thermally Responsive Artificial Muscle for Self-Powered Temperature–Strain Dual-Parameter Sensor

Hyeon Jun Sim [1], Jun Ho Noh [1,2], Jin Hyeong Choi [1] and Changsoon Choi [1,3,*]

[1] Department of Energy and Materials Engineering, Dongguk University, Seoul 04620, Republic of Korea
[2] Department of Advanced Battery Convergence Engineering, Dongguk University, Seoul 04620, Republic of Korea
[3] Research Center for Photoenergy Harvesting & Conversion Technology (phct), Dongguk University, Seoul 04620, Republic of Korea
* Correspondence: cschoi84@dongguk.edu

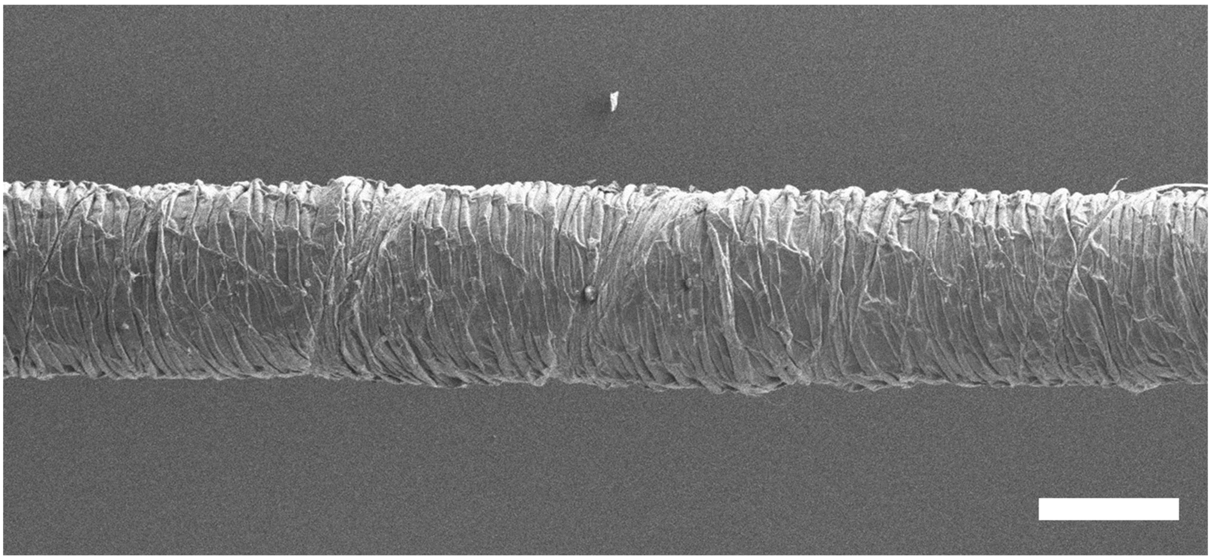

Supplementary Figure S1. The SEM image of MEH fiber (scale bar: 100 μm).

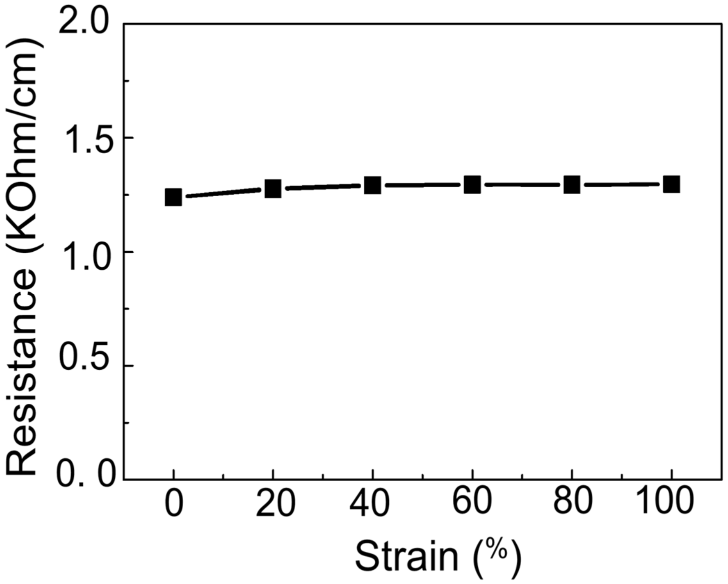

Supplementary Figure S2. The electrical resistance of MEH fiber with strain.

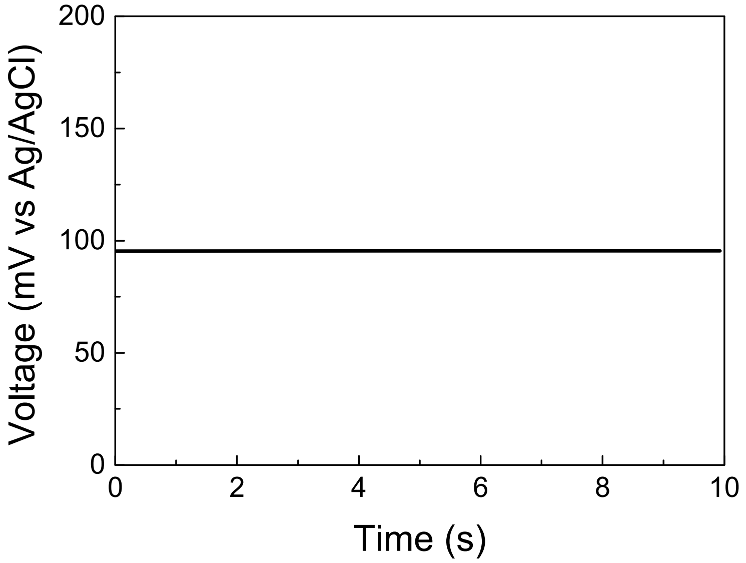

Supplementary Figure S3. The OCV value of MEH fiber when the fiber was immersed in the electrolyte of 0.6M NaCl.

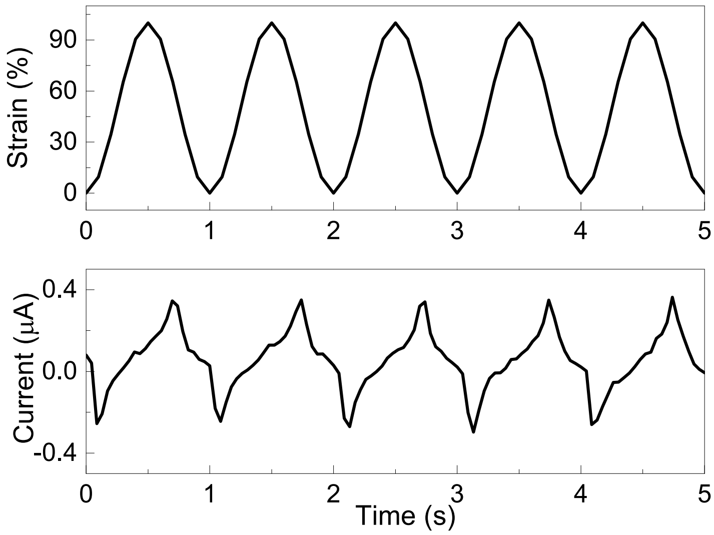

Supplementary Figure S4. Sinusoidally applied tensile strain and resulting in short-circuit current value of MEH fiber when the fiber stretched to 100% strain in the electrolyte of 0.6M NaCl.

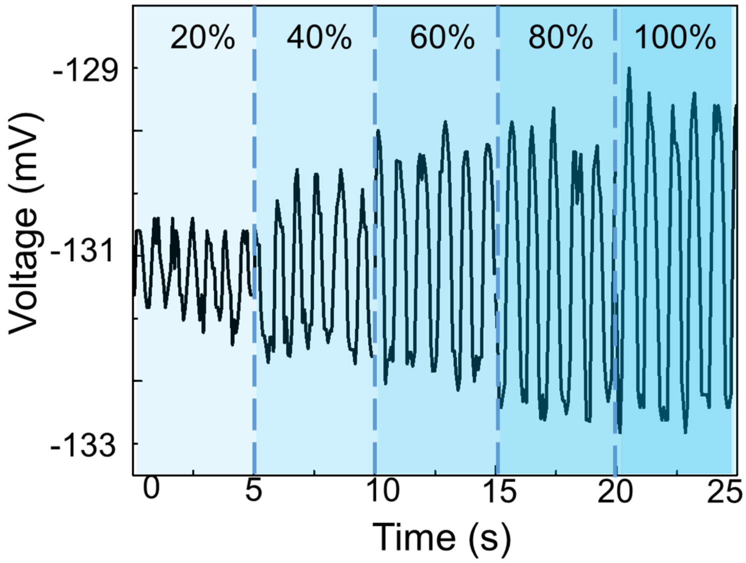

Supplementary Figure S5. The open-circuit voltage with various applied strains from 20% to 100% during the 1-Hz sinusoidal stretch in saline

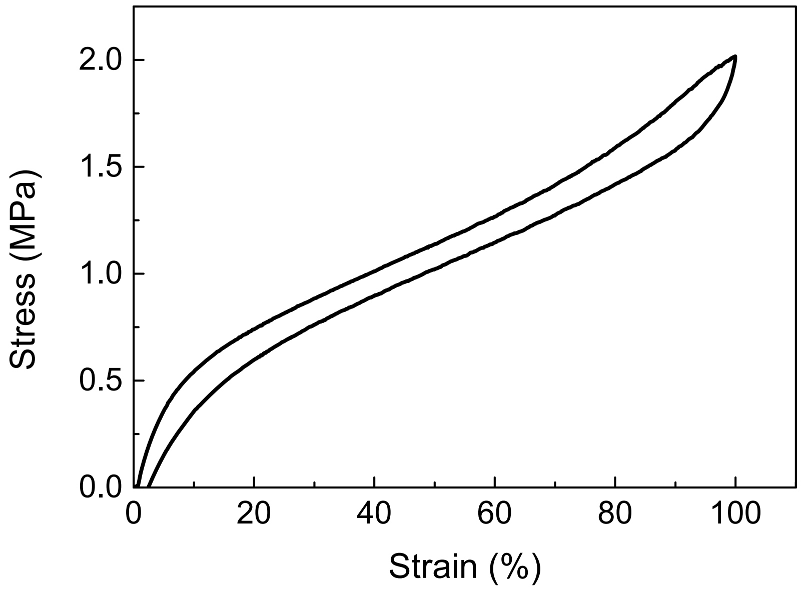

Supplementary Figure S6. The strain-stress curve of MEH fiber

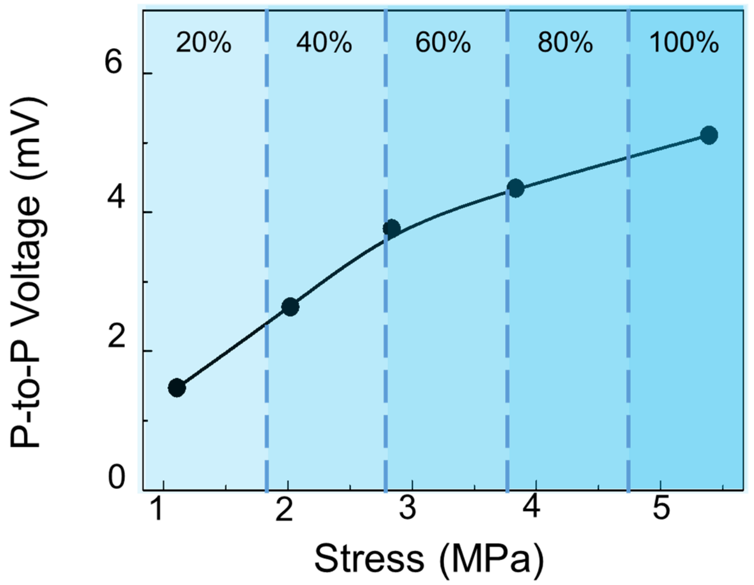

Supplementary Figure S7. The output response of peak-to-peak voltage with applied stress.

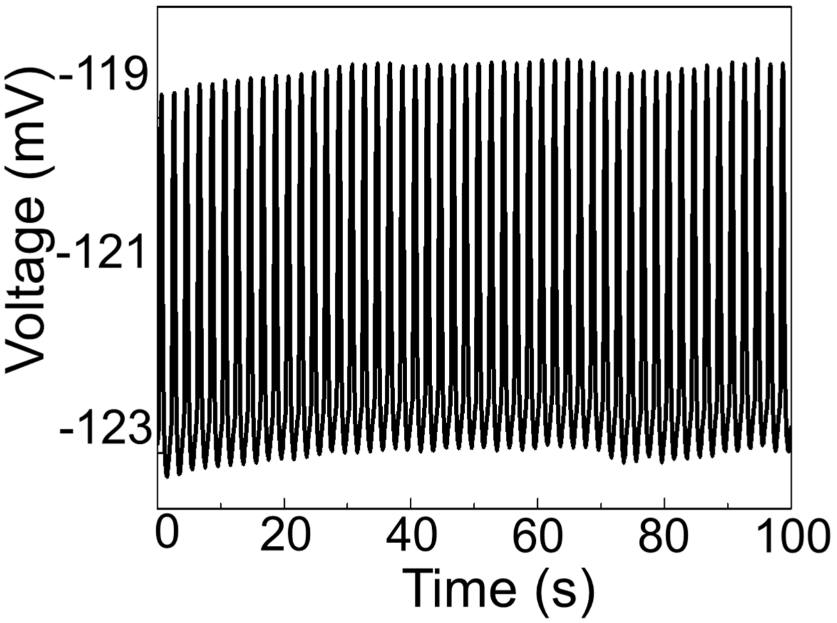

Supplementary Figure S8. The open-circuit voltage output of fiber after 1000 cycles in saline.

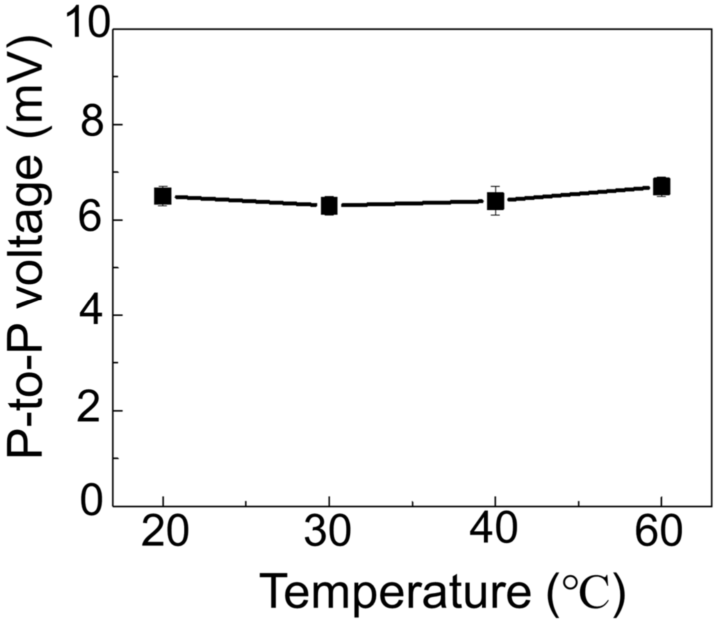

Supplementary Figure S9. The open-circuit peak-to-peak voltage with various environmental temperature from 10 to 40 ℃ during 1-Hz sinusoidal 100% stretch.

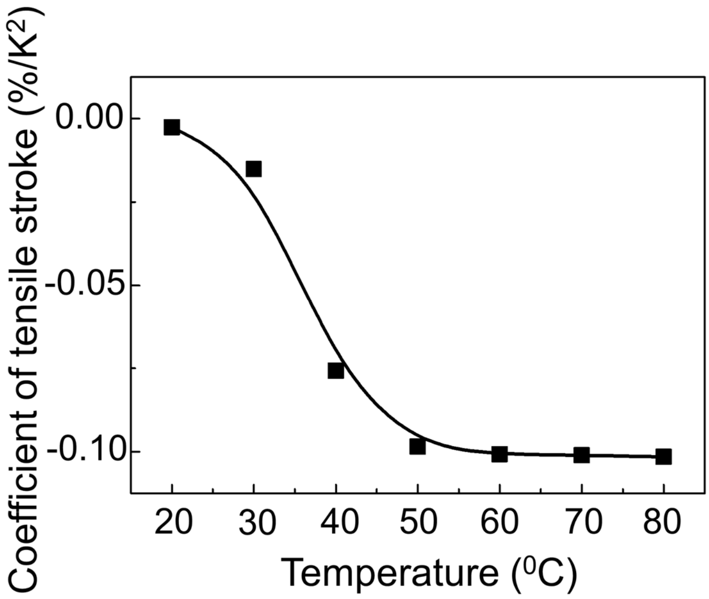

Supplementary Figure S10. the coefficient of tensile stroke with temperature during heating.

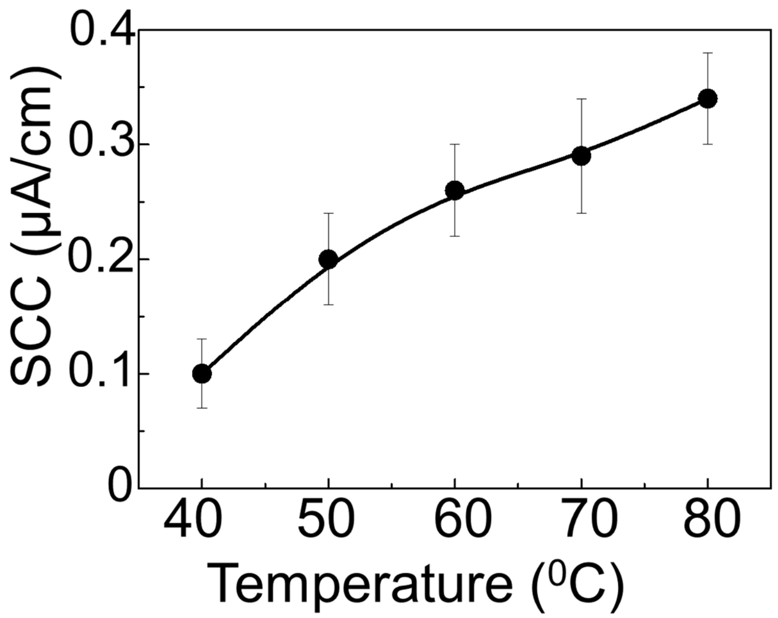

Supplementary Figure S11. the SCC of MEH-TAM with temperature.
